# Circ-0001068 is a novel biomarker for ovarian cancer and inducer of PD1 expression in T cells

**DOI:** 10.18632/aging.103706

**Published:** 2020-10-07

**Authors:** Xinchen Wang, Yingyu Yao, Meiyuan Jin

**Affiliations:** 1 Department of Gynecology and Obstetrics, Tongde Hospital of Zhejiang Province, Hangzhou, China

**Keywords:** exosome, ovarian cancer, circRNA, circ-0001068, miR-28-5p

## Abstract

Ovarian cancer is a primary gynecological malignancy with a global 5-year survival rate of 44%. The majority of patients present with advanced disease at initial diagnosis because of the lack of an effective early detection screening test. Circular RNAs (circRNAs) within exosomes in the circulatory system are effective diagnostic and therapeutic biomarkers for many diseases, especially tumors. In this study, we used microarrays to identify 6 circRNAs that were upregulated and 37 circRNAs that were downregulated in exosomes from ovarian cancer patients as compared to healthy volunteers. We validated the accumulation trends for the 6 upregulated circRNAs in the training set using qRT-PCR and found that circ-0001068 was significantly higher in the serum exosomes from the ovarian cancer patients as than healthy volunteers. Circ-0001068 was next evaluated further in a larger cohort. As with the training set, results from the larger cohort revealed that levels of circ-0001068 in the exosomes were significantly higher in ovarian cancer patients than healthy volunteers. Circ-0001068 was also delivered into T cells and induced PD1 expression by acting as a competing endogenous RNA (ceRNA) for miR-28-5p through the exosomes.

## INTRODUCTION

Ovarian cancer (OC) is recognized as a primary gynecological malignancy with a global 5-year survival rate of 44% [1]. Currently, surgery, including cytoreduction or debulking, and chemotherapy are the main therapeutic options for OC. More than 75% of patients with OC present with advanced disease stages at the initial time of diagnosis because of the asymptomatic nature of OC during the early stages [1]. Because the optimal time to treat patients with OC is during the early stages of the disease, the therapeutic options for patients with advanced OC are limited; and the 5-year survival rate of advanced OC decreases to 25% [1]. Therefore, novel, noninvasive, and effective biomarkers for the early detection of OC are needed.

Liquid biopsy is a novel and minimally invasive blood-based technique that can be used for the early diagnosis of and population screening for OC [2, 3]. Currently, the main liquid biopsy resources are circulating tumor DNA (ctDNA), circulating tumor cells (CTCs), circulating microRNAs, platelets, and exosomes [2, 3]. Exosomes are nanoscale extracellular vesicles that are secreted by almost all kinds of living cells and released into bodily fluids, including blood, urine, saliva, and breast milk [4, 5]. Exosomes contain various nucleic acids, including long non-coding RNAs (lncRNAs), microRNAs (miRNAs), and circular RNAs (circRNAs) [4, 5], and mediate intercellular communication, tumor microenvironment, immune system functions, development and differentiation of cells, cell signaling, and viral replication via the delivery of biomolecules [4].

Circular RNAs are a novel class of endogenous noncoding RNAs that are characterized by back splicing that results in covalently closed loop structures without 5′ caps and 3′ poly tails [6]. CircRNAs are present in almost all kinds of species and are involved in numerous cell functions, such as regulating transcription in the nucleus, functioning as efficient microRNA sponges, competing with pre-mRNA splicing, and serving as circRNA-protein interactions [6]. Recently, several studies have shown that circRNAs were enriched and stable in the exosomes of bodily fluids, and they may serve as potential biomarkers for cancer diagnoses [6].

The aim of the present study was to compare differences in the expression of circRNAs in the serum exosomes between OC patients and healthy volunteers using microarray analyses in order to identify biomarkers for the diagnosis and prognosis of OC. Our results showed that circ-0001068 was significantly increased in the serum exosomes of the OC patients as compared with that in the healthy controls. Additionally, OC cells delivered circ-0001068 into T cells to promote tumor immune invasion.

## RESULTS

### CircRNA expression profiles in the serum exosomes serum from ovarian cancer patients and healthy volunteers

**Figure 1:**
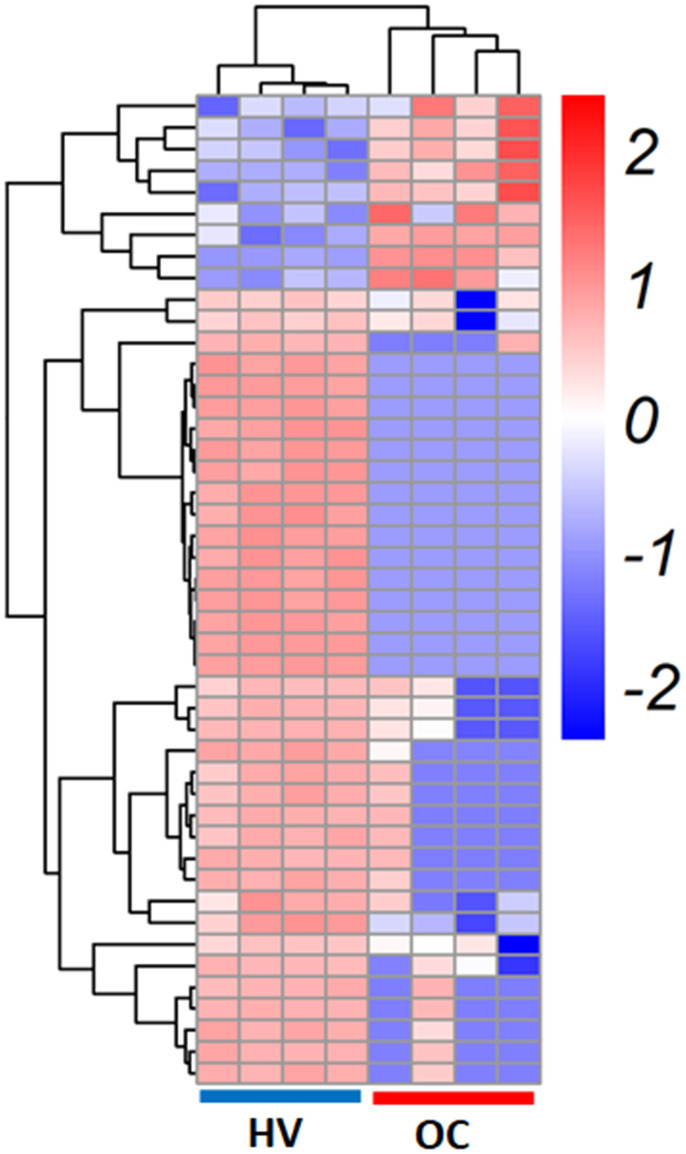
**Identification of circRNAs in the serum exosomes from healthy volunteers (HV) and ovarian cancer (OC) patients.** Heatmap of circRNAs in serum exosomes from HV and OC patients.

High-throughput human circRNA microarray was performed using serum exosomes from 4 OC patients and 4 healthy volunteers to assess circRNA expression profiles. The heat map showed that the profiles of exosomal circRNA were different between OC patients and healthy volunteers (Figure 1). Specifically, 6 circRNAs were upregulated and 37 circRNAs were downregulated in the OC patients as compared with those in the health volunteers (Table 1).

**Table 1:** Significantly upregulated circRNAs in the serum exosomes from ovarian cancer patients.

	**position**	**strand**	**gene symbol**	**Fold Change (OC VS HV)**	***p***
hsa_circ_0001068	chr2:135010665-135012215	+	MGAT5	6.92	0.02
hsa_circ_0000123	chr1:145441364-145441533	+	TXNIP	6.35	0
hsa_circ_0001423	chr4:87967317-87968746	+	AFF1	4.35	0
hsa_circ_0000688	chr16:29845053-29845387	+	MVP	2.33	0.01
hsa_circ_0000284	chr11:33307958-33309057	+	HIPK3	2.17	0.01
hsa_circ_0001523	chr5:124036706-124036962	-	ZNF608	2.09	0.01

### Circ-0001068 is highly expressed in exosomes of serum from OC patients

**Figure 2:**
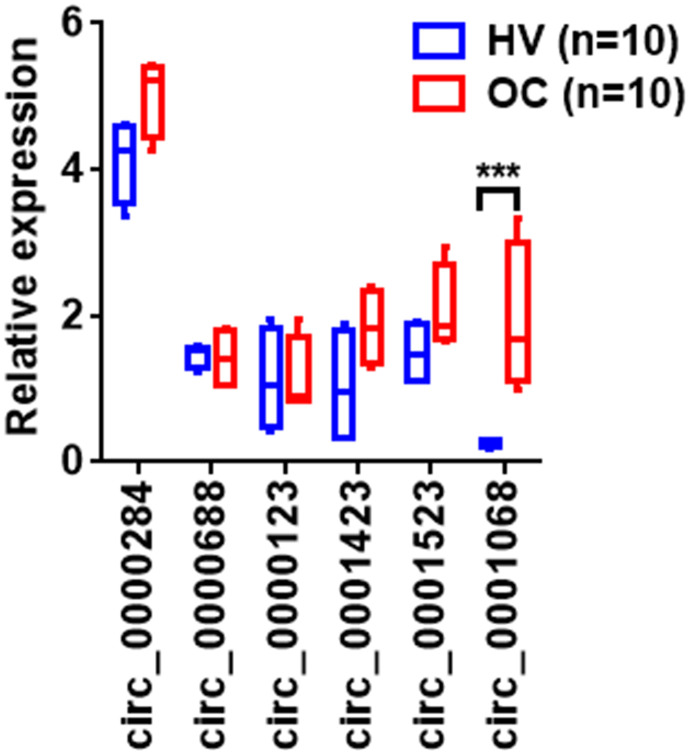
**The relative levels of circRNAs in the serum exosomes from 10 healthy volunteers (HV) and 10 ovarian cancer (OC) patients.** The relative levels of circRNAs in the serum exosomes of the HV and OC patients were determined using qRT- PCR. ***P < 0.001.

**Figure 3:**
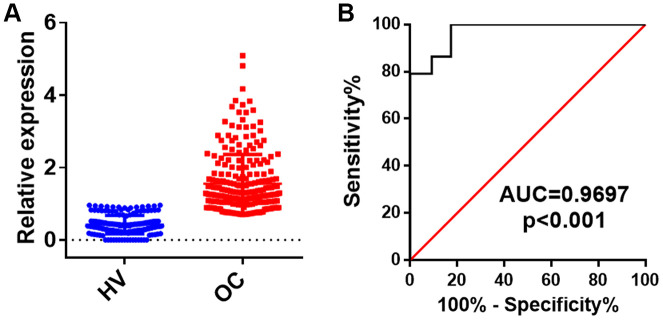
**The relative levels and ROC analysis of circ-0001068 in the exosomes of serum from healthy volunteers (HV) and ovarian cancer (OC) patients.** (**A**) The relative levels of circ-0001068 in the exosomes of serum from 43 healthy volunteers (HV) and 85 ovarian cancer patients (OC). (**B**) the ROC analysis of circ-0001068. ***P < 0.001.

CircRNAs that exhibited a ≥ 2-fold increase (*p* < 0.05) in OC patients as compared with those in healthy volunteers were used as biomarkers. Consequently, 6 upregulated circRNAs were selected for further validation in two independent cohorts (53 HV and 95 OC) using RT-qPCR assays (Table 1). The concentrations of the 6 upregulated circRNAs were measured in a training set that included 10 OC patients and 10 healthy volunteers. The results showed that the concentration of circ-0001068 was significantly increased in the serum exosomes from the OC patients as compared with that in the serum exosomes from the healthy volunteers (Figure 2); however, there were no differences in the concentrations of the other five circRNAs between the OC patients and healthy volunteers. Next, we evaluated the concentration of circ-0001068 in a larger cohort that included 43 healthy volunteers and 85 OC patients. Similar to the results from the training set, the results from the larger cohort revealed that the expression level of circ-0001068 was significantly upregulated in the OC patients as compared with that in the healthy volunteers (Figure 3A). Subsequently, a receiver operating characteristic (ROC) curve analysis was performed to investigate the diagnostic value of circ-0001068 for OC, and the area under the curve (AUC) of circ-0001068 was 0.9697 (95% confidence interval [CI], 0.9619-0.9852). These results suggest that circ-0001068 in serum exosomes may serve as a noninvasive biomarker for OC.

### Exosomes derived from ovarian cancer cells deliver circ-0001068 into T cells

**Figure 4:**
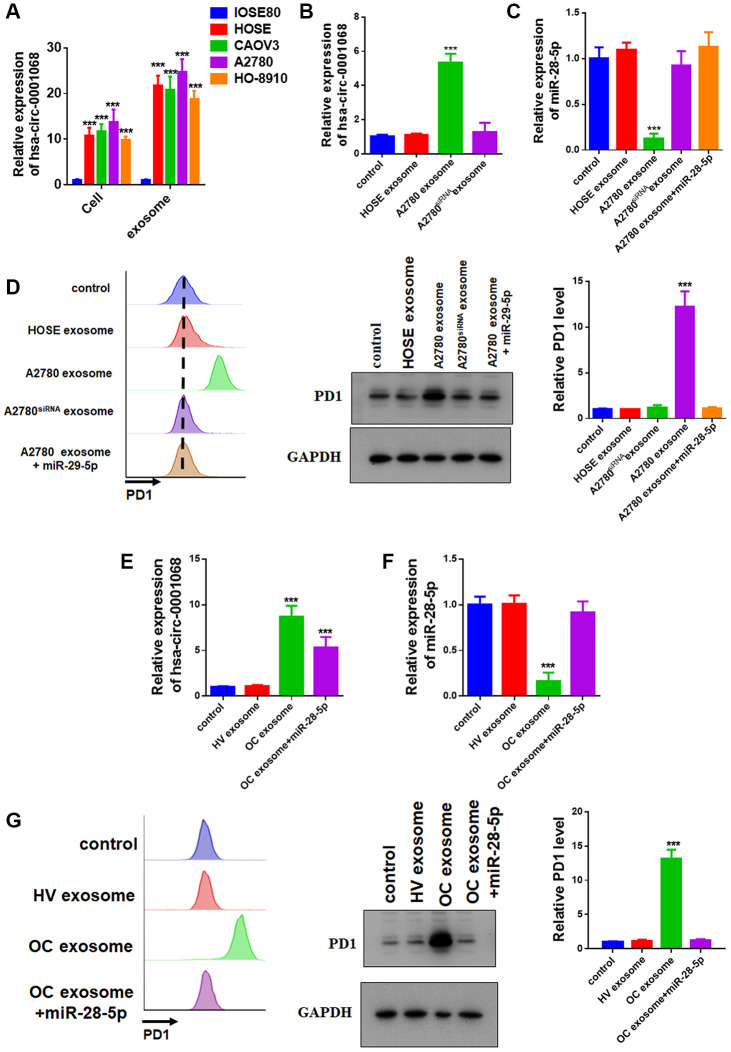
**Ovarian cancer cells deliver circ-0001068 into T cells to induce PD1 expression by sponging miR-28-5p through exosomes.** (**A**) The relative levels of circ-0001068 in the exosomes from the ovarian surface epithelium (OSE) cell line, (IOSE80) and ovarian cancer cell lines (HOSE, CAOV3, A2780, and HO-8910). (**B**) The relative levels of circ-0001068 in the Jurkat T cells that were incubated with exosomes from HOSE cells, A2780 cells, and A2780 cells that were transfected with circ-0001068 siRNA. (**C**) The relative levels of miR-28-5p in the Jurkat T cells that were incubated with exosomes from HOSE cells, A2780 cells, A2780 cells that were transfected with circ-0001068 siRNA, and A2780 cells plus an miR-28-5p mimic. (**D**) Flow cytometry was used to determine the relative levels of PD1 in the Jurkat T cells that were incubated with exosomes from HOSE cells, A2780 cells, A2780 cells that were transfected with circ-0001068 siRNA, and A2780 cells plus an miR-28-5p mimic. Right: representative image; Left: quantitative analysis. (**E**) The relative levels of circ-0001068 in the Jurkat T cells that were incubated with serum exosomes from healthy volunteers, ovarian cancer patients, and ovarian cancer patients plus an miR-28-5p mimic. (**F**) The relative levels of miR-28-5p in the Jurkat T cells that were incubated with serum exosomes from healthy volunteers, ovarian cancer patients, and ovarian cancer patients plus an miR-28-5p mimic. (**G**) The relative levels of PD1 in the Jurkat T cells that were incubated with serum exosomes from healthy volunteers, ovarian cancer patients, and ovarian cancer patients plus an miR-28-5p mimic. Right: representative image; Left: quantitative analysis. ***P < 0.001.

Exosomes that are present in the circulatory system are mainly derived from blood cells, including platelets, red blood cells, and peripheral blood mononuclear cells [7]. However, tumor cells can also secrete exosomes into circulation and deliver certain intracellular components, including circRNAs, into T lymphocytes that alter T cell function [8]. We compared the expression level of circ-0001068 in the normal ovarian surface epithelium cell line, IOSE80, and OC cell lines, including HOSE, CAOV3, A2780, and HO-8910, and discovered that circ-0001068 was significantly upregulated in OC cell lines, especially A2780, as compared with that in the IOSE80 cells (Figure 4A). We also compared differences in the expression level of circ-0001068 between 20 paired OC tumor tissues and distal normal tissues and found that circ-0001068 was significantly increased in OC tumor tissues as compared with that in the distal normal tissues (Supplementary Figure 1A). Similarly, circ-0001068 was also enriched in the exosomes from the OC lines as compared with that in the IOSE80 cells (Figure 4A). Therefore, we incubated the exosomes that were secreted from the IOSE80 cells or A2780 cells with the Jurkat T cells and discovered that the expression level of circ-0001068 was increased in the Jurkat T cells that were incubated with the exosomes from the A2780 cells as compared with that in the Jurkat T cells that were incubated with exosomes from the IOSE80 cells. Next, we downregulated circ-0001068 in the A2780 cells by transfecting the A2780 cells with circ-0001068 siRNA (Supplementary Figure 2A) and found that the expression level of circ-0001068 in the exosomes of A2780 cells transfected with circ-0001068 siRNA was decreased, and the increased level of circ-0001068 in the Jurkat T cells was attenuated (Figure 4B).

### circ-0001068 in the exosomes derived from ovarian cancer cells induce PD1 expression by sponging miR-28-5p

In order to determine the function of circ-0001068 in T cells, we performed bioinformatics analysis to identify the miRNAs that interact with circ-0001068 in T cells. As shown in Supplementary Figure 2B, circ-0001068 binds to two sites on miR-28-5p. Luciferase reporter assays were used to confirm whether miR-28-5p directly targeted circ-0001068 through the binding sites. Although miR-28-5p repressed the luciferase activity of the circ-0001068 wild-type reporter plasmid, it had no effect on the luciferase activity of the circ-0001068 mutant reporter (the seed sequence was mutated from AGUUCCU to UCAAGGA) in the Jurkat T cells (Supplementary Figure 2C, 2D). These results suggest that circ-0001068 may be a ceRNA for miR-28-5p in Jurkat T cells. In accordance with the results, we found that the expression levels of miR-28-5p were significantly decreased in the Jurkat T cells that were incubated with the exosomes from the A2780 cells as compared with those that were incubated with exosomes from the IOSE80 cells (Figure 4C). Moreover, this decrease was abolished by co-transfection with miR-28-5p (Figure 4C). CircRNAs elevate downstream target expression through sponging miRNAs, and studies have reported that miR-28-5p repressed the expression of PD1 in T cells to induce T cell exhaustion (Supplementary Figure 2E) [9]. We further investigated the influence of circ-0001068 on the PD1 expression in T cells by flow cytometry and found that the expression level of PD1 was significantly increased in T cells that were incubated with exosomes from the A2780 cells as compared with that in T cells that were incubated with exosomes from the IOSE80 cells (Figure 4D). Moreover, this increase was abolished following co-transfection with an miR-28-5p mimic (Figure 4D), which upregulated the expression level of miR-28-5p in T cells (Supplementary Figure 2C). In summary, we found that circ-0001068 in the exosomes from OC cells induced PD1 expression by sponging miR-28-5p,

### Serum exosomes from ovarian cancer patients induce PD1 expression in T cells

Then, we incubated the exosomes that were isolated from OC patients or healthy volunteers with the Jurkat T cells and discovered that the expression level of circ-0001068 was significantly upregulated in the T cells that were incubated with exosomes from OC patients as compared with that in the T cells that were incubated with the exosomes from healthy volunteers. Consistently, this increase also prominently decreased the expression level of miR-28-5p, which resulted in the upregulation of PD1 in the T cells (Figure 4G).

## DISCUSSION

OC is among the most cancerous tumors in the female reproductive system [1]. The mortality rate of OC has continued to increase over the past ten years [1]. At the first time of diagnosis, more than 75% of women have advanced disease; therefore, the therapeutic options for the majority of patients are limited. The 5-years survival rate decreases from 90% in the early stages to 25% in the advanced stages; however, the current medical treatments for OC are very limited, especially for OC with metastasis. Thus, novel noninvasive and effective biomarkers for the early detection of OC are needed.

Currently, CA125 and HE4 are the most widely used biomarkers for OC diagnosis, and they have been approved by the US Food and Drug Administration (FDA) [10]. However, both CA125 and HE4 are usually used as aids to monitor tumor progression or recurrence in OC cases instead of early detection markers because of their relatively low sensitivity and specificity [10]. For example, CA125 was only increased in 50% of patients with stage I disease; however, it was elevated in other gynecological cancers, benign gynecological diseases, and non-gynecological malignancies [11–13]. Recently, a number of studies confirmed the RNAs in the exosomes of the peritoneal blood were closely correlated with tumor progression [14, 15] Additionally, exosomes, particularly the RNAs in exosomes, may serve as tumor-specific biomarkers [7]. CircRNAs are a novel class of endogenous noncoding RNAs with a closed loop structure. Studies have reported that circRNAs are enriched and stable in exosomes in the circulation system and function as molecular biomarkers for tumor diagnosis [6]. In this study, we found that circ-0001068 in the serum exosomes from OC patients was significantly upregulated as compared with that in the serum exosomes from healthy volunteers, and the AUC of circ-0001068 was 0.9697 (95% confidence interval [CI], 0.9619-0.9852). These results indicate that circ-0001068 in exosomes may serve as a noninvasive biomarker for OC. However, larger cohorts are need to confirm this finding.

Exosomes are 30–150 nm nanoscale extracellular vesicles that are shed by almost all kinds of tumor cells and play multiple roles in tumor progression, including cell proliferation [16, 17], drug resistance [18], angiogenesis and metastasis [19], immune modulation [20, 21], and premetastatic niche formation [22]. Exosomes secreted by tumors cells also contain tumor specific biomolecules, such as proteins, lipids, microRNAs, mRNAs, long noncoding RNAs, and circRNAs, and exosomes deliver these biomolecules into immune cells to promote tumor immune evasion (i.e., T cells that induce T cell exhaustion) [8]. In our study, we found that exosomes from OC cells delivered circ-0001068 into T cells to induce PD1 expression, and this resulted in T cell exhaustion. As a novel class of competing endogenous RNAs (ceRNAs), circRNAs play a complicated regulatory role in cancer development and progression [23]. Importantly, an increasing number of studies have confirmed that circRNAs function as miRNA sponges to modulate the function of miRNA targets during the progression of OC. For example, the circRNA, circ-ITCH, sponges miR-145 to suppress the progression of ovarian carcinoma [24]. Additionally, hsa_circ_0061140 sponges miR-370 to upregulate the miR-370 target gene, FOXM1, which promotes the growth and metastasis of OC [25]. In our study, we found that circ-0001068 was delivered into T cells and sponged miR-28-5p. Moreover, circ-0001068 relieved the inhibitory effect of miR-28-5p on its target gene, PD1, to induce PD1 expression in T cells.

Overall, we revealed that circ-0001068 was significantly upregulated in the exosomes of serum from OC patients as compared with that in healthy volunteers. We also confirmed circ-0001068 that was secreted by OC cells was delivered into T cells and sponged miR-28-5p, which resulted in the increased expression of PD1 and T cell exhaustion. Our results demonstrate that circ-0001068 may serve as novel noninvasive biomarker and target for the diagnosis and treatment OC.

## MATERIALS AND METHODS

### Patients’ characteristics

Twenty pairs of OC tissues and adjacent non-cancerous tissues were collected from patients who were diagnosed with OC at the Tongde Hospital of Zhejiang Province. The serum samples from 95 OC patients who were not receiving any treatments and 53 matched healthy volunteers were collected at the Tongde Hospital of Zhejiang Province. The patient characteristics and clinical features are summarized in Table 2. This study was approved by the Ethics Committee of Tongde Hospital of Zhejiang Province, and written informed consent was obtained from all patients. All experiments were performed in accordance with relevant guidelines and regulations. Protocols were designed and performed according to the principles of the Declaration of Helsinki.

**Table 2:** Patient characteristics and clinical features.

		**Ovarian Cancer tissue (n=20)**	**Normal serum (n=53)**	**Ovarian Cancer serum (n=95)**
Age		56.6+8.7	58.2±12.5	57.1±9.8
Stage				
	I	4		16
	II	11		55
	III	5		22
	IV	0		2
T				
	1	4		25
	2	12		57
	3	4		10
	4	0		3
N				
	0	13		46
	1	4		31
	2	3		13
	3	0		5
M				
	0	20		93
	1	0		2

### Exosome isolation and exosome RNA extraction

The Total Exosome Isolation Reagent (from serum) (Cat#4478360, Invitrogen, USA) and Total Exosome Isolation Reagent (from cell culture media) (Cat#4478359, Invitrogen, USA) were used to isolate exosomes from the serum and cell culture media according to the manufacturer’s protocol. Total RNAs were extracted from exosomes using the mirVana PARIS Kit (Ambion, Thermo Scientific, Shanghai, China) according to the manufacturer's protocol. Synthetic *Caenorhabditis elegans* miRNA cel-miR-39 (5’-UCACCGGGUGUAAAUCAGCUUG-3’), (RiboBio, Guangzhou, China) was used for normalization [26]. Finally, a NanoDrop was used to quantify the total RNA, and the expression of the circRNAs was determined using Arraystar microarray analysis.

### Quantitative real-time PCR

A hydrolysis probe–based RT-qPCR assay was performed for exosomal circRNAs and miRNAs according to the manufacturer's protocol. The primers used for circRNAs were synthesized by GenScript (Nanjing, China) and are listed in Supplementary Table 1. The primers used for miRNAs were purchased from Applied Biosystems. All reactions were performed in triplicate, and the data were analyzed using the comparative C_T_ method; cel-miR-39 was used as the exogenous control.

### Exosome incubation with Jurkat T cells and PD1 detection

Jurkat T cells were seeded onto 12-well dishes, and 500 μg of exosomes were added into each well. After incubation for 24 h, the Jurkat T cells were collected for qRT-PCR or flow cytometry analysis. The Jurkat T cells were stained with the PD1 antibody (BioLegend, Clone EH12.2H7), and the expression of PD1 was determined by flow cytometry. Data were analyzed with FlowJo-10 software.

### Luciferase reporter system

The Circular RNA Interactome database (https://circinteractome.nia.nih.gov) was used to predict the miRNAs that interacted with circ-0001068 and miRNAs [27]. The psiCHECK-2 luciferase reporter plasmid containing the wild-type (WT) or mutant type (Mut) of circ-0001068 was purchased from GenScript (Nanjing, China). The Jurkat T cells were co-transfected with miR-28-5p mimics or scramble RNA together with WT circ-0001068 or Mut circ-0001068 luciferase reporter plasmids using Lipofectamine 3000. The luciferase intensity was detected after 48 h using a dual-luciferase reporter assay kit (Promega, USA).

### Statistical analysis

Statistical analysis was performed with SPSS 16.0 software. Student’s t-tests or two-sided χ2 tests were used to compare differences in the variables between patients with OC and healthy volunteers. P < 0.05 was considered statistically significant. A receiver operating characteristic curve (ROC) analysis was performed to estimate the diagnostic value of circRNAs in the exosomes.

## Supplementary Material

Supplementary Figures

Supplementary Table 1
