# Supplementary material for: Circ-0001068 is a novel biomarker for ovarian cancer and inducer of PD1 expression in T cells
Source: Aging (Albany NY). 2020 Oct 7;12(19):19095–106. doi: 10.18632/aging.103706 (PMC7732319; doi:10.18632/aging.103706)
Supplement: Supplementary Figures [file aging-12-103706-s001..pdf]

SUPPLEMENTARY FIGURES

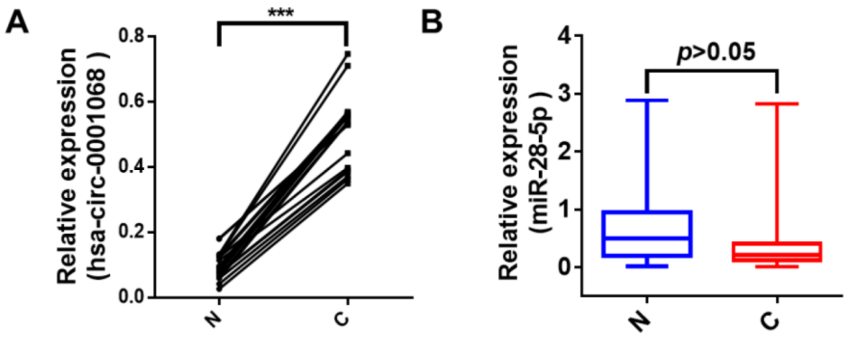

Supplementary Figure 1. The relative levels of circ-0001068 in ovarian cancer tissues (**A**) and the relative levels of miR-28-5p in the serum exosomes (**B**) by qRT- PCR. N: non-cancerous tissues; C: ovarian cancer tissues. ***P < 0.001.

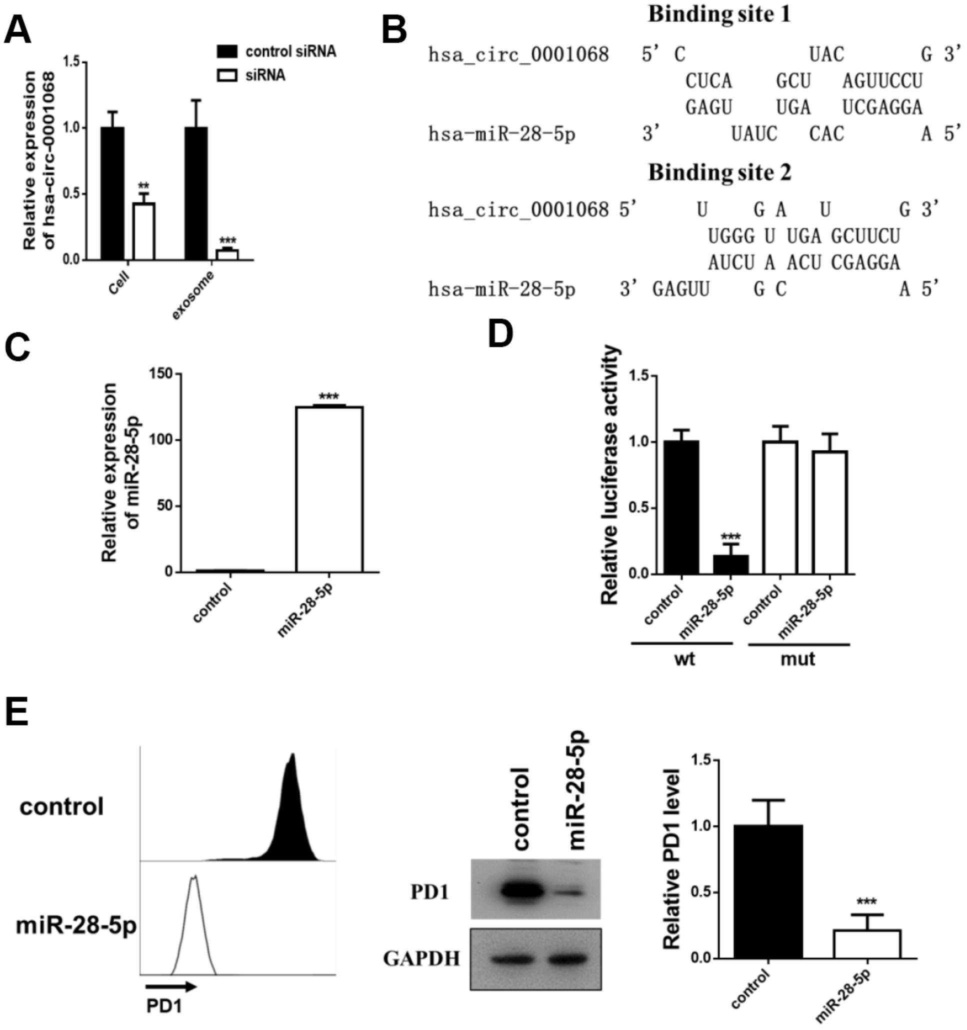

Supplementary Figure 2. miR-28-5p suppress PD1 expression in T cells. (**A**) The efficiencies of siRNA of circ-0001068. (**B**) The interaction between circ-0001068 and miR-28-5p. (**C**) The relative levels of miR-28-5p in the Jurkat cells transfected with scramble RNA and miR-28-5p mimic. (**D**) The luciferase activity in the Jurkat cells transfected with wild type circ-0001068 luciferase report plasmid plus scramble RNA (control), wild type circ-0001068 luciferase report plasmid plus miR-28-5p mimic (miR-28-5p), muted circ-0001068 luciferase report plasmid plus scramble RNA (control), and muted circ-0001068 luciferase report plasmid plus miR-28-5p mimic (miR-28-5p). (**E**) The expression level of PD1 in the Jurkat cells transfected with scramble RNA and miR-28-5p mimic by flow cytometry and western blotting. ***P < 0.001.
