# Supplementary material for: Circ-0001068 is a novel biomarker for ovarian cancer and inducer of PD1 expression in T cells
Source: Aging (Albany NY). 2020 Oct 7;12(19):19095–106. doi: 10.18632/aging.103706 (PMC7732319; doi:10.18632/aging.103706)
Supplement: Supplementary Table 1 [file aging-12-103706-s002..pdf]

SUPPLEMENTARY TABLE

Supplementary Table 1. The primers used for qRT-PCR.

CircRNA	Forward(5'-3')	Reserves(5'-3')
**hsa_circ_0001068**	GAGCAGATCCTGGACCTCAG	CCCTGGATCACTGTTGGTCT
**hsa_circ_0000123**	AGAAAAACTCAGGCCCATCC	CATCCAACAAACACCCCTGT
**hsa_circ_0001423**	CGGAGGACTATCGACAGCAG	TGGTTGCGTCTTTCCTTCTC
**hsa_circ_0000688**	AGATCATTCAGGCCACCATC	CCACCACCTTGTCTCCATCT
**hsa_circ_0000284**	GGCAGCCTTACAGGGTTAAA	GGGTAGACCAAGACTTGTGAGG
**hsa_circ_0001523**	CCTGGGACCAGTGTGAATTT	TGGATGAAGATGGGGAAAAG
